# Vascular Endothelial Growth Factor Receptor Inhibitors in Chinese Patients With Advanced Radioactive Iodine-Refractory Differentiated Thyroid Cancer: A Network Meta-Analysis and Cost-Effectiveness Analysis

**DOI:** 10.3389/fendo.2022.909333

**Published:** 2022-07-14

**Authors:** Youwen Zhu, Kun Liu, Kailing Wang, Libo Peng

**Affiliations:** ^1^ Department of Oncology, Xiangya Hospital, Central South University, Changsha, China; ^2^ Department of Gastroenterology, Xiangya Hospital, Central South University, Changsha, China; ^3^ Department of Oncology, Loudi Central Hospital, The Central Hospital of Loudi Affiliated to the University of South China, Loudi, China

**Keywords:** advanced radioactive iodine-refractory differentiated thyroid cancer, apatinib, lenvatinib, cost-effectiveness analysis, network meta-analysis

## Abstract

**Introduction:**

Two targeted drugs (apatinib and lenvatinib) show clinical efficacy in first-line treatment of Chinese patients with radioactive advanced iodine-refractory differentiated thyroid cancer (RAIR-DTC) and are recommended by the Chinese Society of Clinical Oncology guidelines. Considering the high clinical cost of long-term vascular endothelial growth factor receptor inhibitor administration and to determine which of the two targeted drugs is preferable, we opted to conduct a cost-effectiveness analysis (CEA) and network meta-analysis (NMA).

**Material and Methods:**

The results of NMA and CEA included in the two phase III randomized clinical trials REALITY (NCT03048877) and Study-308 (NCT02966093), in which Bayesian NMA and CEA were performed on 243 and 149 Chinese patients, respectively, were retrieved. Overall survival and progression-free survival (PFS) for apatinib versus lenvatinib were determined by NMA. CEA involved the development of a 20-year Markov model to obtain the total cost and quality-adjusted life-years (QALYs), and this was followed by sensitivity and subgroup analyses.

**Results:**

Compared with lenvatinib, apatinib therapy provided a 0.837 improvement in QALY and $6,975 reduction in costs. The hazard ratio of apatinib versus lenvatinib and the cost of the targeted drugs had a significant impact on the model. According to the sensitivity analysis, apatinib was more cost-effective and had no correlation with willingness-to-pay in China. Subgroup analysis showed that apatinib maintained PFS more economically.

**Conclusion:**

NMA and CEA demonstrated that apatinib was more cost-effective compared to lenvatinib in the first-line treatment of Chinese RAIR-DTC patients.

## Introduction

Thyroid cancer (TC) is the tenth most common cancer worldwide, with more than 580,000 new cases diagnosed and more than 43,000 deaths (1). More than 190,000 new cases have been reported in China (1). Differentiated TC (DTC) is the most prevalent, accounting for more than 90% of all TCs (2). The probability of recurrence or metastasis disease was close to 60% (3). Radioactive iodine therapy was the primary treatment for patients with advanced DTC, but 30% of patients become radioactive iodine-refractory (RAIR) cancers (4, 5), which have a 10-year survival rate of 10% (6).

Two phase III trials, DECISION (NCT00984282) and SELECT (NCT01321554), demonstrated that sorafenib and lenvatinib extend progression-free survival (PFS) by a significant 10.3 months and 18.3 months, respectively, compared with placebo in the whole population of RAIR-DTC patients (7, 8). Subsequently, sorafenib and lenvatinib have been approved by Chinese Society of Clinical Oncology (CSCO) and were added to their guidelines in 2017 and 2020, respectively, as standard treatments for Chinese patients with RAIR-DTC (9). Unfortunately, no significant increase in overall survival (OS) was observed in either study, which included populations from multiple countries (7, 8). Therefore, as China is a country of major RAIR-DTC prevalence, the limited treatment regimens cannot meet the demand. Treatment strategies for Chinese patients with RAIR-DTC need to be improved and widely applied in clinical practice.

Apatinib is a small-molecule angiogenic inhibitor of vascular endothelial growth factor receptor (VEGFR)-2 of high selectivity. Lenvatinib is a multitargeted tyrosine kinase inhibitor (TKI) specific for VEGFR-1, -2, and -3. Based on Chinese people with RAIR-DTC, two studies showed the significant clinical benefits of TKIs. The REALITY trial (NCT03048877) showed that apatinib significantly extended the median PFS (22.2 months; HR, 0.26; 95% CI, 0.14–0.47; P < 0.001) and OS (HR, 0.42; 95% CI, 0.18–0.97; P = 0.04) of Chinese patients in advanced stages of RAIR-DTC compared with placebo (10). Study-308 (NCT02966093) demonstrated that lenvatinib significantly improved the median PFS (23.9 months; HR, 0.16; 95% CI, 0.10–0.26; P < 0.0001) of Chinese patients with RAIR-DTC compared with placebo. However, there was no significant benefit in terms of OS (HR, 0.42; 95% CI, 0.18–0.97; P = 0.04) (11). Because of these findings, both apatinib and lenvatinib are recommended as advanced RAIR-DTC treatment in the 2021 CSCO guidelines (9). With the remarkable results of the two Chinese-patient-based studies, the concomitant cost-effectiveness of the two TKI types has become the focus of attention. To answer this question, we compared the cost-effectiveness of apatinib and lenvatinib for patients with advanced RAIR-DTC from the perspective of Chinese payers.

## Methods

Network meta-analysis and cost-effectiveness analysis (NMA and CEA) were guided by the PRISMA NMA checklist and the Economic Assessment Report Standard Statement (CHEERS) checklist, respectively (**Supplementary Material eTables 1**, **2**).

### Network Meta-Analysis

#### Study Selection and Assessment of Bias Risks

We searched PubMed, Embase, Cochrane, and Web of Science for compliant English-language publications up to March 15, 2022, with the search terms “PD-1”, “tyrosine kinase inhibitor”, “vascular endothelial growth factor receptor inhibitors”, “apatinib”, “lenvatinib”, “radioactive iodine-refractory differentiated thyroid cancer”, and “clinical trial” (**Supplementary Material eTable 3**). Abstracts from meetings of the American Society of Clinical Oncology (ASCO) and the European Society of Medical Oncology (ESMO) were also reviewed. The eligible literature met the following criteria: (1) Phase III randomized controlled trials; (2) apatinib or lenvatinib were compared for Chinese patients with advanced RAIR-DTC; (3) the outcomes were OS and PFS; (4) details of treatment strategies and treatment-related adverse events (AEs) were included. Those not meeting the inclusion criteria were disregarded. Two reviewers (Y.W.Z. and K.L.) independently screened the selected studies to avoid missing articles and extract relevant data. The bias risk assessment for these clinical trials was performed using Cochrane RevMan (version 5.4, available: https://training.cochrane.org).

#### Statistical Analysis

We used R software (version 4.1.1, available: http://www.rproject.org) and software package “netmeta” for Bayesian network meta-analysis to obtain the HRs of OS and PFS for apatinib versus lenvatinib. However, due to the lack of data from the two studies that can provide information for assessing the heterogeneity between the tests, we chose the method of Su et al. and adopted the fixed-effect model for analysis (12).

### Cost-Effectiveness Analysis

#### Patients and Treatments

The model patient cohorts from REALITY and Study-308 were used to form a patient simulation cohort. The REALITY trial enrolled 46 Chinese patients with advanced RAIR-DTC who received apatinib treatment from February 17, 2017, with the data expiration date of March 25, 2020. Study-308 enrolled 103 Chinese patients with advanced RAIR-DTC who received lenvatinib treatment from January 11, 2017, with a data cut-off date of July 31, 2019. The baseline characteristics of patients and details of medications used are presented separately in **Supplementary Material** **eTables 4** and **5**. We assumed that the included Chinese patients were 60 years old, 65 kg in weight, 164 cm in height, and 1.72 m^2^ in body surface area (13). Patients receiving apatinib and lenvatinib were assessed with computed tomography or magnetic resonance imaging every 2 and 8 weeks, respectively (10, 11). After progression, all patients received best supportive care (BSC). Finally, each patient who died was given terminal care.

#### Model Construction

A Markov model was developed using TreeAge (Version TreeAge Pro 2021, https://www.treeage.com) to evaluate the cost-effectiveness of apatinib versus lenvatinib for Chinese patients with RAIR-DTC. The Markov model included three health states: PFS, disease progression (PD), and death (**Supplementary Material** **eFigure 2**). The clinical trial treatment protocol and follow-up protocol were applied for 2 months, and when more than 99% of patients died, the time horizon was 20 years. We extracted survival curves from REALITY and Study-30 through GetData (Version 2.26; http://www.getdata-graph-digitizer.com/index.php) and used the survival curves to extract the transition probabilities. Then, the best fitting parameter model was selected from Weibull, Gompertz, exponential, log-logistic, and log-normal distribution using the Akaike information criterion and Bayesian information criterion. After selecting the Weibull fitting parameter model, two parameters were calculated with R software: Scale (λ) and Shape (γ). More details are shown in **Supplementary Material** **eFigure 3** and **eTable 6**. Our main results were total cost, quality-adjusted life-years (QALYs), which is a number derived from a weighted analysis of the quality and annual health discount rates associated with a patient’s annual health status, and incremental cost‐effectiveness ratio (ICER), and we used a willing-to-pay (WTP) threshold of $37,653/QALY (thrice China’s per-capita gross domestic product 2021) to determine cost-effectiveness. When ICER < WTP, the increased cost is completely worth it, and the treatment option have cost-effectiveness; When ICER > WTP, the increased cost is not worth it, and the intervention measures are not cost-effective.

#### Utility and Cost Estimates

As health utility values were reported in the two studies, the published literature was adopted, assuming that the PFS status and PD status had utility scores of 0.80 and 0.50, respectively (14, 15). The disutility of AEs was also considered (16, 17) (**Table 1**).

**Table 1:** Model parameters: baseline values, ranges, and distributions for the sensitivity analysis.

Parameters	Baseline value	Range		Reference	Distribution
		Minimum	Maximum		
Weibull survival model of apatinib					
OS	Scale= 0.0006137, Shape= 1.8950428	–	–	(10)	–
PFS	Scale= 0.003379, Shape= 1.821018	–	–	(10)	–
Weibull survival model of lenvatinib					
OS	Scale= 0.005721, Shape= 1.283299	–	–	(11)	–
PFS	Scale= 0.022440, Shape= 1.152276	–	–	(11)	–
HR for apatinib vs lenvatinib					
OS	0.50	0.16	1.57	Network meta-analysis	–
PFS	1.63	0.75	3.57	Network meta-analysis	–
Risk for main AEs in apatinib group					
Risk of hypertension	0.348	0.277	0.415	(10)	Beta
Risk of hand-foot syndrome	0.174	0.139	0.209	(10)	Beta
Risk of proteinuria	0.152	0.121	0.182	(10)	Beta
Risk of diarrhoea	0.152	0.121	0.182	(10)	Beta
Risk of hypocalcaemia	0.065	0.052	0.078	(10)	Beta
Risk for main AEs in lenvatinib group					
Risk of hypertension	0.621	0.497	0.745	(11)	Beta
Risk of proteinuria	0.233	0.186	0.280	(11)	Beta
Risk of hand-foot syndrome	0.097	0.078	0.116	(11)	Beta
Risk of diarrhoea	0.068	0.054	0.082	(11)	Beta
Risk of platelet count decreased	0.068	0.054	0.082	(11)	Beta
Utility					
PFS	0.80	0.64	0.96	(14, 15)	Beta
PD	0.50	0.40	0.60	(14, 15)	Beta
Disutility					
Platelet count decreased	0.020	0.016	0.024	(16)	Beta
Hand-foot syndrome	0.016	0.013	0.019	(17)	Beta
Diarrhoea	0.014	0.011	0.017	(17)	Beta
Hypertension	0	NA	NA	(17)	Beta
Hypocalcaemia	-^a^	–	–	–	–
Proteinuria	-^a^	–	–	–	–
Drug cost, $/per cycle					
Apatinib	1,850	1,480	2,220	Local Charge	Gamma
Lenvatinib	5,725	4,580	6,870	Local Charge	Gamma
Cost of AEs, $					
Apatinib	10	8	12	(12, 21)	Gamma
Lenvatinib	50	40	60	(12, 16, 21)	Gamma
Administration per cycle	36	29	43	(18)	Gamma
Best supportive care per cycle	440	352	528	(18)	Gamma
Terminal care per patient	2129	1,703	2,555	(18)	Gamma
Tumor imaging per cycle	145	116	174	(19)	Gamma
Laboratory per cycle	232	186	278	(20)	Gamma
Body surface area (meters^2^ )	1.72	1.38	2.06	(13)	Normal
Discount rate	0.03	0	0.05	(22)	Uniform

a The disutilities with regard to hypocalcaemia and proteinuria were not reported.

OS, overall survival; PFS, progression-free survival; AEs, adverse events.

We only considered direct medical costs, including drug treatment, administration, BSC, terminal care, laboratory, tumor imaging, and treatment-related AEs. Drug prices were sourced from Xiangya Hospital of Central South University in China. All remaining costs were derived from the published literature (12, 16, 18–21). All prices are expressed in US dollars, using the exchange rate *$1 =￥6.3389* (March 14, 2022). Based on our consumer price index and a discount rate of 3% per year, healthcare-related costs were inflated to 2022 values in China (22) (**Table 1**).

#### Sensitivity Analyses

Sensitivity analyses were applied to resolve uncertainties in the model. One-way sensitivity analysis included relevant parameters and 20% variation ranges, and the probability sensitivity analysis involved 10,000 Monte Carlo simulations to obtain an acceptable curve (22).

We included subgroups of patients separated by age, sex, and pathological typing for analysis. We first performed network meta-analysis to obtain the HRs of existing subgroups of PFS (apatinib versus lenvatinib). Then, according to the method adopted by Dong et al., the ICER and cost-effectiveness probability of each subgroup were obtained (22).

## Results

### Network Meta-Analysis

A total of 299 records were identified by searching major literature databases, and we eventually included two phase III randomized clinical trials (REALITY and Study-308) based on the criteria, with a total of 243 Chinese patients with advanced RAIR-DTC (**Supplementary Material eFigures 1**, **4**). In the REALITY trial, 92 patients received either apatinib or placebo. In the Study-308 trial, 151 patients were treated with lenvatinib or placebo (**Supplementary Material eTable 7**). NMA showed that the HRs of OS and PFS for apatinib compared with lenvatinib were 0.50 (95% CI, 0.16–1.57) and 1.63 (95% CI, 0.75–3.51), respectively (**Table 1**).

### Cost-Effectiveness Analysis

#### Base-Case Analyses

For 149 Chinese patients with advanced RAIR-DTC, apatinib gained 5.905 QALYs at a total cost of $85,551. Apatinib regimes were accompanied by a relatively small improvement in QALY and lowered healthcare costs by $6,975 compared to lenvatinib. Hence, of the two treatment strategies, apatinib was the most efficacious and cost-effective (**Table 2**).

**Table 2:** Baseline results.

Outcomes	Apatinib	Lenvatinib
QALYs	5.905	5.068
Change in cost, $^a^	0.837	
Total cost $	85,551	92,526
Change in QALYs^a^	-6,975	
ICER $/QALY	Dominated^b^ (-8,333)	
WTP $/QALY	37,653	

a Change in cost and change in QALYs represent the results of apatinib minus lenvatinib.

b Apatinib showed higher effectiveness and lower cost, as compared with the lenvatinib.

ICER, incremental cost-effectiveness ratio; LY, life-year; QALY, quality-adjusted life-year; WTP, willingness-to-pay.

#### Sensitivity Analyses

**Figure 1:**
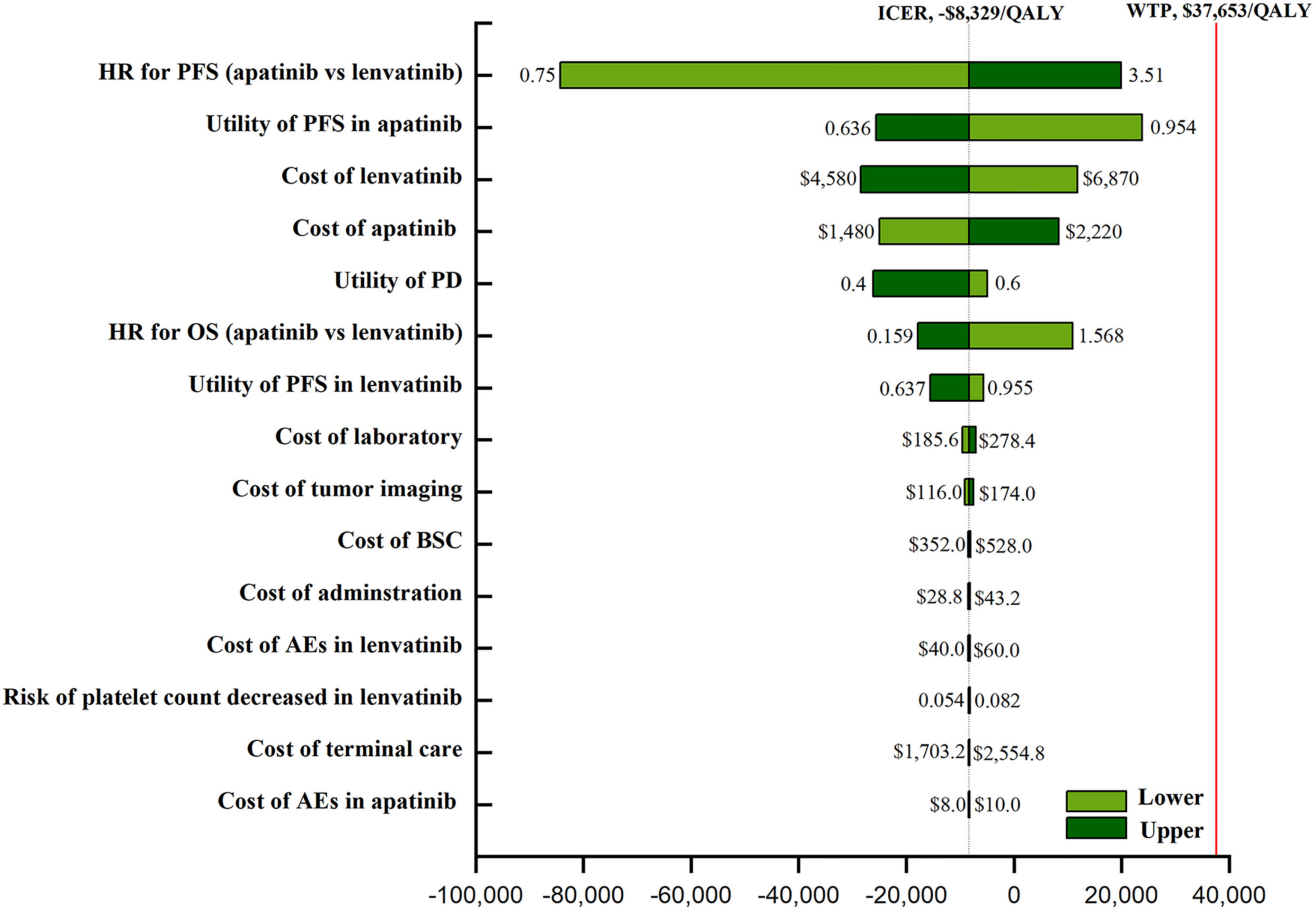
The one-way sensitivity analyses of the apatinib vs Lenvatinib. PFS, progression-free survival; PD, disease progression; OS, overall survival; BSC, best supportive care; AEs, adverse events.

**Figure 2:**
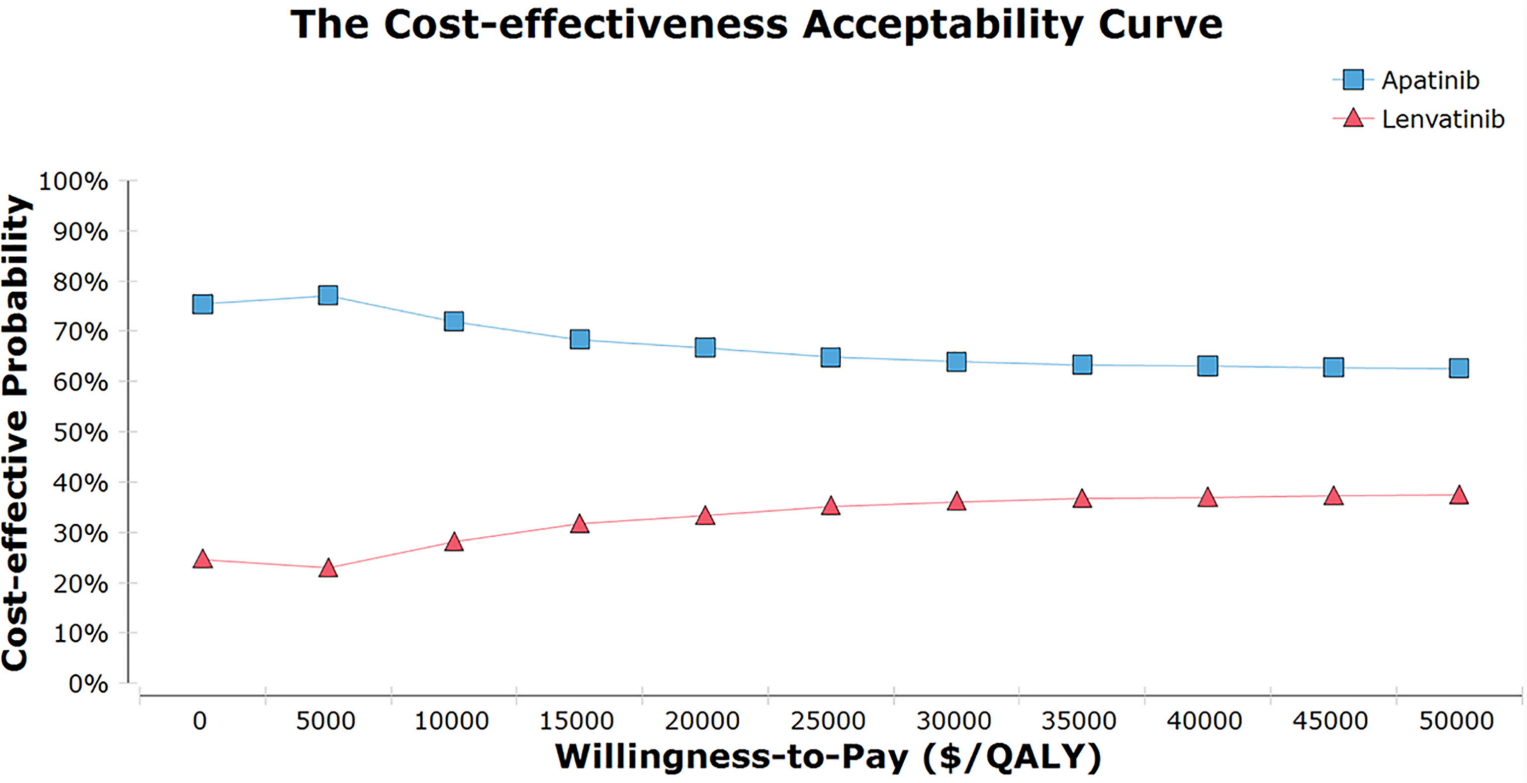
The cost-effectiveness acceptability curves for the apatinib vs Lenvatinib. QALY, quality-adjusted life-year.

One-way sensitivity analysis indicated that the HR for PFS (apatinib versus lenvatinib), the utility of PFS with apatinib, and the cost of TKIs were sensitivity factors for the model. The incidence of AEs had negligible effect (**Figure 1**). The cost-effectiveness acceptability curve demonstrated that the apatinib strategy was consistently cost-effective, regardless of WTP (**Figure 2**). Among all the included subgroups, apatinib performed better in prolonging survival, with an increase in the QALYs for apatinib versus lenvatinib ranging from 0.746 to 1.002. Apatinib showed dominant cost-effectiveness for a subgroup of patients that were ≤65 years of age, male, and had papillary TC (PTC) (**Supplementary Material eTable 8**).

## Discussion

TC is endocrine system malignancy (23). The morbidity, mortality, and burden of the disease continue to rise worldwide (24–26). China has one of the highest TC burdens in the world, with an average cost of $11,560 per patient in the first year after diagnosis (27, 28). Because of rising healthcare costs, treatment cost evaluations are necessary. TKI is currently on the radar of clinicians and patients with RAIR-DTC. Two previous studies, DECISION (NCT00984282) and SELECT (NCT01321554), demonstrated the significant clinical efficacy of TKIs (7, 8). Therefore, three economic assessments have been published based on two major studies. Huang et al. and Tremblay et al. showed that the ICER generated by lenvatinib and sorafenib in patients with advanced RAIR-DTC were $103,925 per QALY and $95,695 per QALY, respectively, and that lenvatinib is cost-effective compared with sorafenib at a WTP of $150,000 per QALY and $100,000 per QALY, respectively, from the US perspective (29, 30). Leslie et al. showed lenvatinib to be more cost-effective than sorafenib (ICER = $25,275 per QALY) or placebo (ICER = $40,869 per QALY) and that sorafenib was also cost-effective compared to placebo (ICER = $64,067 per QALY) (15). The final analysis demonstrated lenvatinib to be the most cost-effective option for RAIR-DTC at a WTP of 100,000 per QALY, although both lenvatinib and sorafenib were more cost-effective than placebo (15). These studies evaluated the cost performance of the two TKIs based on the perspective of US patients and obtained consistent results that show lenvatinib may be more cost-effective. With the development of new drugs, Chinese physicians and patients are gradually paying attention to the cost and efficacy of TKIs. Therefore, using the Markov model and the clinical efficacy and safety data from two large, randomized phase III clinical trials, we estimated the cost-effectiveness over a 20-year time horizon for apatinib and lenvatinib as therapies for RAIR-DTC. This study of RAIR-DTC patients in China showed that apatinib therapy provided a 0.837 improvement in QALY and $6,975 reduction in costs compared with lenvatinib, resulting in ICER value is definitely lower than the WTP value. Deep meaning indicates that apatinib is a superior treatment strategy compared to lenvatinib, achieving higher efficacy as well as a lower healthcare cost. Therefore, apatinib was more cost-effective compared to lenvatinib in the first-line treatment of Chinese RAIR-DTC patients. Although, it is worth considering that the additional costs associated with lenvatinib are mainly due to follow-up, meaning that the same follow-up plan should be set up for the same cancer type.

We used sensitivity analysis to confirm model uncertainty. From the one-way sensitivity analysis, we deduced that the model’s most influential parameter was the HR of PFS (apatinib versus lenvatinib), underscoring the need for robust head-to-head clinical data. It was also sensitive to the utility of PFS, and the analysis found that, for patients with a lower utility of PFS, apatinib had a more favorable economic outcomes compared with lenvatinib, but apatinib had a worse economic outcome for patients with a higher utility. Another important influencing variable was the price of the TKIs: apatinib’s price increase of more than 56% and lenvatinib’s price decrease of more than 46% mean lenvatinib is more cost-effective. Changing other parameters had virtually no influence on our results, and due to the high cost of TKIs, reducing the prices of apatinib and lenvatinib was considered the most practical measure in the context of cost-effectiveness and optimal logistics.

The results were consistent with baseline in the subgroup analysis, showing that the ICER (apatinib versus lenvatinib) was lower than the WTP. In the sensitivity analysis, apatinib was more cost-effective than lenvatinib 55%. It is worth noting that in the subgroups of Chinese patients of ≤65 years, male, and PTC, apatinib generation was associated with a higher efficacy at a lower cost. Changes in the QALYs of PFS in PTC were widely observed, leading to the possibility that TKI was more effective among many pathological types of PTC. This is consistent with the results of two previous retrospective studies, the multivariate analysis of which identified histological grade as a favorable prognostic factor. Lars et al. reviewed 173 patients with PTC, and their analysis showed that the 10-year survival rates of patients with low-and high-grade PTC were 95.3% and 75.1%, respectively (31). Allen et al. reviewed 37,858 cases of PTC and found the prognosis of moderately differentiated and poorly differentiated PTC was positively correlated with OS compared with highly differentiated PTC (HR, 1.21; 95% CI, 1.04-1.41; P = 0.02 and HR, 2.62; 95%CI, 2.23-3.08, P < 0.001) (32). Therefore, under the influence of cost-effectiveness, pathological typing to predict treatment prognosis needs to be performed.

In China, those approving innovative drugs to maintain our healthcare system should not only take into account the huge clinical and economic benefits but also realize the importance of prognostic marker biomarker analysis. Unfortunately, we did not analyze predictive markers. However, studies have found that thyroglobulin (Tg) and anti-thyroglobulin (TgAb) are important prognostic factors for guiding clinical treatment and are valuable parameters for long-term monitoring of DTC patients. Patients with Tg of <0.2 ng/mL or thyroid-stimulating hormone-stimulated Tg of <1 ng/mL responded well to treatment and had minimal levels of recurrence and an almost complete absence of disease-specific deaths (33–36). However, patients with higher than normal Tg levels (inhibition of Tg of ≥1 ng/mL or stimulation of Tg of ≥10 ng/mL) or elevated TgAb values after treatment had a low mortality rate, but a significant proportion of this group developed structural disease recurrence (33, 37). A prospective study involving serum from 249 patients showed that VEGF and VEGFR may have prognostic value for RAIR-DTC (38). Levels of VEGF showed a clear link to a lower risk of recurrence (overall response [OR], 0.08; 95% CI, 0.01-1.43; P = 0.018 and OR, 0.08; 95% CI, 0.01-1.37; P = 0.016) (38). In addition, molecular markers such as BRAF, EGFR, Ki67, and P53 were also potentially effective prognostic factors (39–42).

This study had several significant advantages. Firstly, we ascertained the cost-effectiveness of two TKI-based RAIA-DTC treatments over a 20-year time range using an economic model. As input for our model, clinical efficacy and safety data were extracted from high-quality phase III clinical trial datasets, and the costs were from the perspective of Chinese payers. We concluded that our model provides long-term cost and effectiveness predictions that are easily translatable to clinical practice. Secondly, we considered the disutility generated by AEs. We used the average health utility of PFS in patients with advanced RAIR-DTC and corrected it using the disutility generated by AEs. When evaluating the economic benefits of the two TKIs, only the negative utility generated by severe AEs had a strong correlation with the quality of life (QoL) (43). Finally, we compared the cost-effectiveness of the two TKIs and added three subgroups that might be useful in clinical practice.

The study had some limitations. Firstly, the survival data were obtained from the interim analysis results of the phase III clinical trials REALITY and Study-308. The survival data will mature with the extension of follow-up time, and the model will become more stable. Secondly, these two TKI schemes were not directly evaluated in any of the trials. Therefore, we compared the two TKIs schemes indirectly using the NMA findings from two phase III clinical trials with similar research content and characteristics of the included patients. However, this method comes with potential uncertainties. Thirdly, to simplify the calculation, we assumed that patients in both groups only received BSC after treatment with TKIs for recurrence, so the analysis may have underestimated the cost of PD. However, we discovered from the sensitivity analysis that the economic burden of PD had little effect on the model’s outcomes. Fourthly, since neither the REALITY nor Study-308 reports provided QoL data, the health state utility in this model was obtained from previously published data and was based on patients in the US or UK. As the QoL of Chinese patients with RAIR-DTC has not yet been reported, this was an essential deviation. Including the QoL of Chinese patients in future studies means the economic results will be updated in time. Fifthly, because of the lack of subgroup survival curves in both studies, we were unable to run a complete model for each subgroup, and the original group equilibrium generated by NMA analysis may not apply to the subgroups. Therefore, the results of the subgroup analysis should be interpreted with caution. Finally, in this model, we only considered the cost and corresponding disutility of treatment-related grade 3/4 and ≥5% AEs, which may have had some influence on the overall cost and utility. However, sensitivity analysis showed that the incidence and disutility of major AEs had little effect on the results.

## Conclusions

In this network meta-analysis and cost-effectiveness analysis, apatinib was a more desirable treatment strategy than lenvatinib for Chinese patients with RAIR-DTC at any WTP threshold. Innovative therapies that provide significant results are pivotal, and lowering the prices of these drugs is critical to achieving their cost-effectiveness. Therefore, apatinib presents a new treatment option with an optimal cost-effective ratio for RAIR-DTC patients.

## Data Availability Statement

The original contributions presented in the study are included in the article/**Supplementary Material**. Further inquiries can be directed to the corresponding author.

## Author Contributions

YZ, KL, KW, and LP performed the experiments. YZ and KL analyzed the data. LP contributed materials and analysis tools. YZ, KL, KW, and LP wrote the manuscript. YZ and KL contributed equally. YZ, KL, KW, and LP approved the manuscript. All authors contributed to the article and approved the submitted version.

## Conflict of Interest

The authors declare that the research was conducted in the absence of any commercial or financial relationships that could be construed as a potential conflict of interest.

## Publisher’s Note

All claims expressed in this article are solely those of the authors and do not necessarily represent those of their affiliated organizations, or those of the publisher, the editors and the reviewers. Any product that may be evaluated in this article, or claim that may be made by its manufacturer, is not guaranteed or endorsed by the publisher.
