# Supplementary material for: Vascular Endothelial Growth Factor Receptor Inhibitors in Chinese Patients With Advanced Radioactive Iodine-Refractory Differentiated Thyroid Cancer: A Network Meta-Analysis and Cost-Effectiveness Analysis
Source: Front Endocrinol (Lausanne). 2022 Jul 14;13:909333. doi: 10.3389/fendo.2022.909333 (PMC9329872; doi:10.3389/fendo.2022.909333)
Supplement: Supplementary file 1 [file DataSheet_1.pdf]

eFigure 1. Study Selection

eFigure 2. Model Structure for Cost-effectiveness Analysis.

eFigure 3. Kaplan-Meier Curve Fitting and Extrapolation.

eFigure 4. Model of Network Meta-analysis.

eFigure 5. Risk of Bias Summary.

eTable 1. PRISMA NMA Checklist.

eTable 2. CHEERS Checklist.

eTable 3. Search Strategy.

eTable 4. Drug Dose and Cost.

eTable 5. Patient Baseline Characteristics.

eTable 6. Summary of Statistical Goodness-of-fit of Kaplan-Meier Curve.

eTable 7. Characteristics of RCTs Included in the Study.

eTable 8. Results of subgroup analyses.

eFigure 1. Study Selection.

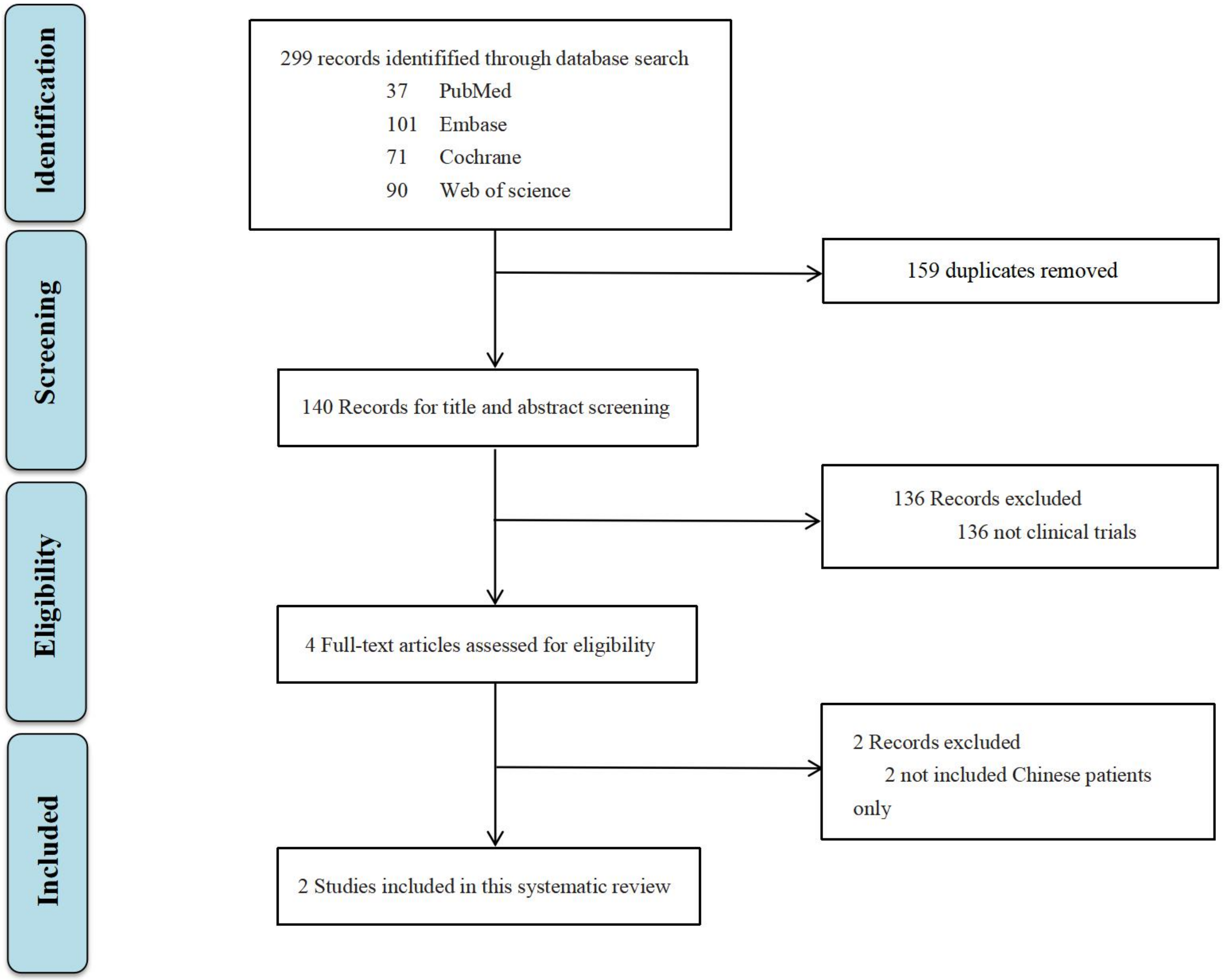

eFigure 2. Model Structure for Cost-effectiveness Analysis.

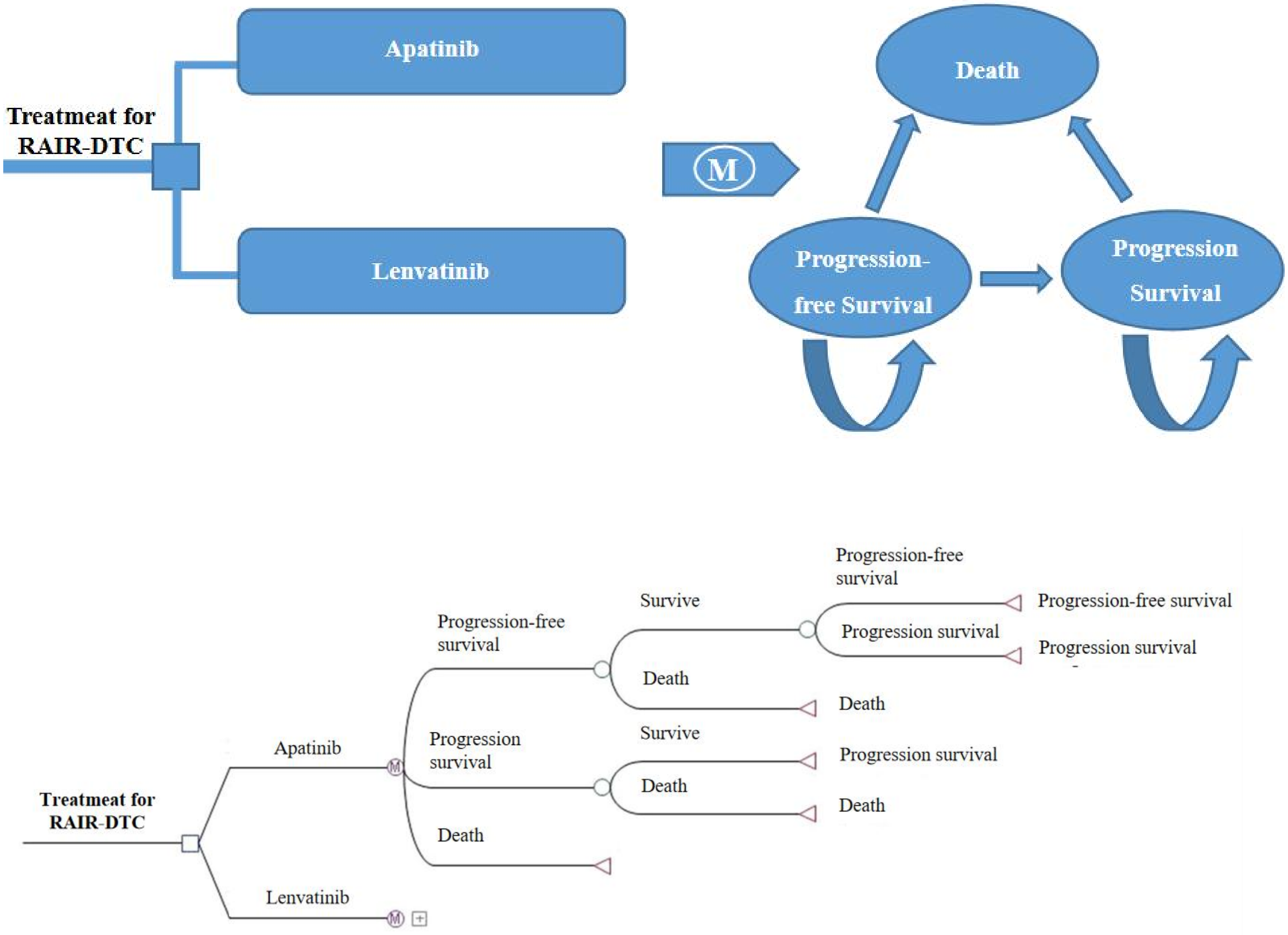

Abbreviation: RAIR-DTC, radioactive iodine-refractory differentiated thyroid cancer; M, Markov.

eFigure 3. Kaplan-Meier Curve Fitting and Extrapolation.

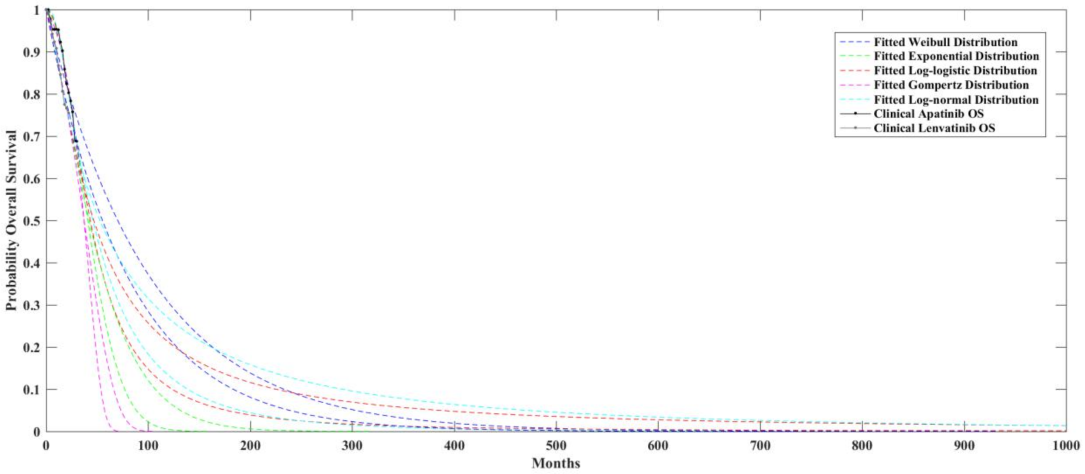

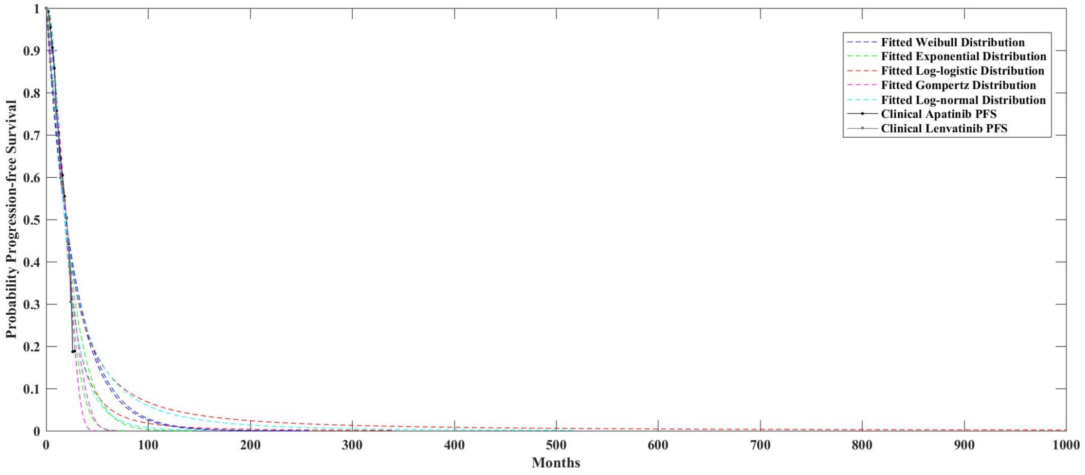

Abbreviation: OS, overall survival; PFS, progression-free survival.

eFigure 4. Model of Network Meta-analysis.

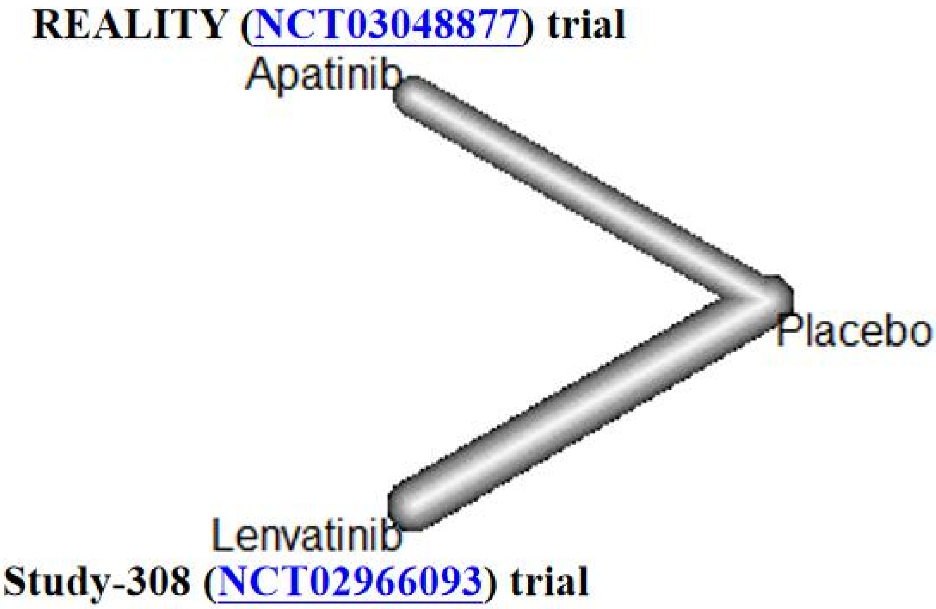

eFigure 5. Risk of Bias Summary.

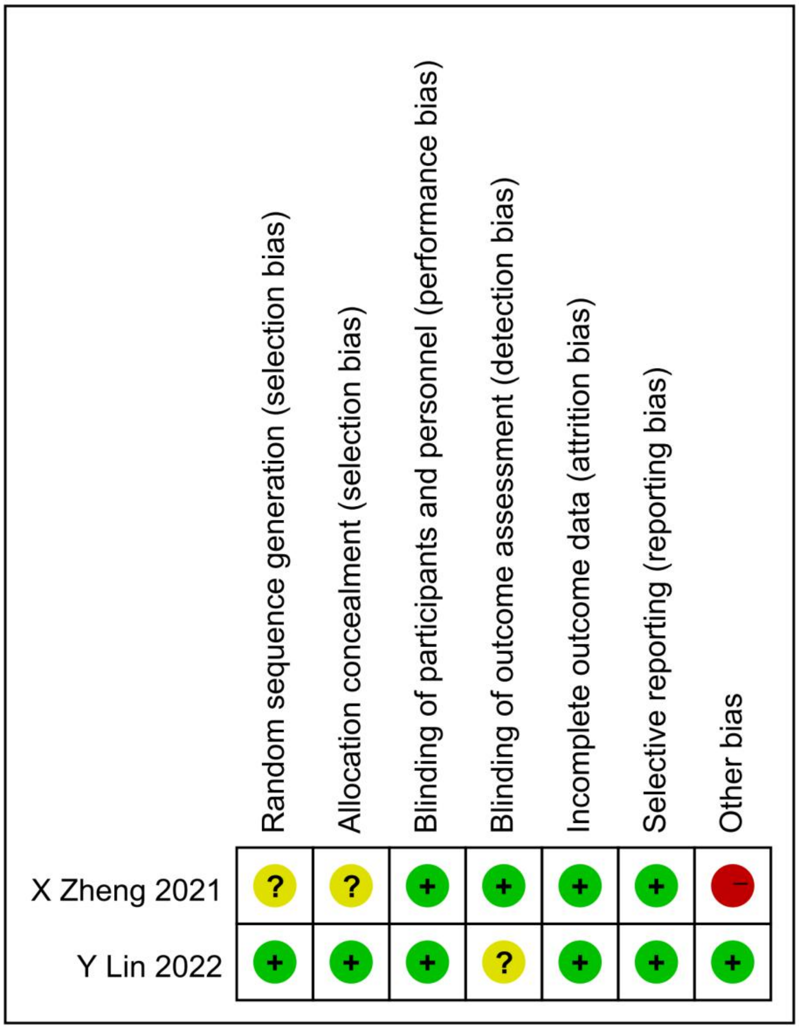

Firstly, the REALITY (Y Lin) and Study-308 (X Zheng) were double-blind clinical trials, which mean patients and investigators were masked. Secondly, the REALITY trial was judged at low risk in random sequence generation and allocation concealment due to assign patients via the minimization randomization method through randomization and trial supply management. The Study-308 trial was judged at unclear risk because of not expounding the details of random sequence generation. Thirdly, the REALITY was judged at unclear risk on account of on details of blinding of outcome assessment. The Study 308 trial was judged at low risk because of independent imaging review. In addition, they were at low risk in the reporting bias given both had research proposals and reported all the predetermined outcome indicators (primary and secondary outcomes). Lastly, regarding other bias, because the results may be skewed to the better because the clinical trial was sponsored by corporations, the study-308 trial was judged at high risk and the REALITY trial at low risk.

eTable 1. PRISMA NMA Checklist.

Section/Topic	Item #	Checklist Item	Reported on Page #
**TITLE**			
Title	1	Identify the report as a systematic review *incorporating a network meta-analysis (or related form of meta-analysis).*	1
**ABSTRACT**			
Structured summary	2	Provide a structured summary including, as applicable:<br>**Background:** main objectives<br>**Methods:** data sources; study eligibility criteria, participants, and interventions; study appraisal; and *synthesis methods, such as network meta-analysis.*<br>**Results:** number of studies and participants identified; summary estimates with corresponding confidence/credible intervals; *treatment rankings may also be discussed. Authors may choose to summarize pairwise comparisons against a chosen treatment included in their analyses for brevity.*<br>**Discussion/Conclusions:** limitations; conclusions and implications of findings.<br>**Other:** primary source of funding; systematic review registration number with registry name.	2
**INTRODUCTION**			
Rationale	3	Describe the rationale for the review in the context of what is already known*, including mention of why a network meta-analysis has been conducted.*	3-4
Objectives	4	Provide an explicit statement of questions being addressed, with reference to participants, interventions, comparisons, outcomes, and study design (PICOS).	4
**METHODS**			
Protocol and registration	5	Indicate whether a review protocol exists and if and where it can be accessed (e.g., Web address); and, if available, provide registration information, including registration number.	Not applicable
Eligibility criteria	6	Specify study characteristics (e.g., PICOS, length of follow-up) and report characteristics (e.g., years considered, language, publication status) used as criteria for eligibility, giving rationale. *Clearly describe eligible treatments included in the treatment network, and note whether any have been clustered or merged into the same node (with justification).*	4<br>eTable 3
Information sources	7	Describe all information sources (e.g., databases with dates of coverage, contact with study authors to identify additional studies) in the search and date last searched.	eTable 7
Search	8	Present full electronic search strategy for at least one database, including any limits used, such that it could be repeated.	4<br>eTable 3
Study selection	9	State the process for selecting studies (i.e., screening, eligibility, included in systematic review, and, if applicable,	eFigure 1

		included in the meta-analysis).	
Data collection process	10	Describe method of data extraction from reports (e.g., piloted forms, independently, in duplicate) and any processes for obtaining and confirming data from investigators.	4<br>eFigure 5
Data items	11	List and define all variables for which data were sought (e.g., PICOS, funding sources) and any assumptions and simplifications made.	5<br>Table 1
**Geometry of the network**	**S1**	Describe methods used to explore the geometry of the treatment network under study and potential biases related to it. This should include how the evidence base has been graphically summarized for presentation, and what characteristics were compiled and used to describe the evidence base to readers.	eFigure 4
Risk of bias within individual studies	12	Describe methods used for assessing risk of bias of individual studies (including specification of whether this was done at the study or outcome level), and how this information is to be used in any data synthesis.	eFigure 5
Summary measures	13	State the principal summary measures (e.g., risk ratio, difference in means). *Also describe the use of additional summary measures assessed, such as treatment rankings and surface under the cumulative ranking curve (SUCRA) values, as well as modified approaches used to present summary findings from meta-analyses.*	5
Planned methods of analysis	14	Describe the methods of handling data and combining results of studies for each network meta-analysis. This should include, but not be limited to:<br>• *Handling of multi-arm trials;*<br>• *Selection of variance structure;*<br>• *Selection of prior distributions in Bayesian analyses; and*<br>•*Assessment of model fit.*	5
**Assessment of Inconsistency**	**S2**	Describe the statistical methods used to evaluate the agreement of direct and indirect evidence in the treatment network(s) studied. Describe efforts taken to address its presence when found.	5
Risk of bias across studies	15	Specify any assessment of risk of bias that may affect the cumulative evidence (e.g., publication bias, selective reporting within studies).	eFigure 5
Additional analyses	16	Describe methods of additional analyses if done, indicating which were pre-specified. This may include, but not be limited to, the following:<br>• Sensitivity or subgroup analyses; • Meta-regression analyses;<br>• *Alternative formulations of the treatment network; and* • *Use of alternative prior distributions for Bayesian analyses (if applicable).*	Not applicable

RESULTS†			
Study selection	17	Give numbers of studies screened, assessed for eligibility, and included in the review, with reasons for exclusions at each stage, ideally with a flow diagram.	8<br>eFigure 1
**Presentation of network structure**	**S3**	Provide a network graph of the included studies to enable visualization of the geometry of the treatment network.	eFigure 3
**Summary of network geometry**	**S4**	Provide a brief overview of characteristics of the treatment network. This may include commentary on the abundance of trials and randomized patients for the different interventions and pairwise comparisons in the network, gaps of evidence in the treatment network, and potential biases reflected by the network structure.	7<br>eFigure 1
Study characteristics	18	For each study, present characteristics for which data were extracted (e.g., study size, PICOS, follow-up period) and provide the citations.	7<br>eTable 7
Risk of bias within studies	19	Present data on risk of bias of each study and, if available, any outcome level assessment.	7<br>eFigure 5
Results of individual studies	20	For all outcomes considered (benefits or harms), present, for each study: 1) simple summary data for each intervention group, and 2) effect estimates and confidence intervals. *Modified approaches may be needed to deal with information from larger networks.*	7
Synthesis of results	21	Present results of each meta-analysis done, including confidence/credible intervals. *In larger networks, authors may focus on comparisons versus a particular comparator (e.g. placebo or standard care), with full findings presented in an appendix. League tables and forest plots may be considered to summarize pairwise comparisons.* If additional summary measures were explored (such as treatment rankings), these should also be presented.	7<br>Table 1
**Exploration for inconsistency**	**S5**	Describe results from investigations of inconsistency. This may include such information as measures of model fit to compare consistency and inconsistency models, *P* values from statistical tests, or summary of inconsistency estimates from different parts of the treatment network.	Not applicable
Risk of bias across studies	22	Present results of any assessment of risk of bias across studies for the evidence base being studied.	Not applicable
Results of additional analyses	23	Give results of additional analyses, if done (e.g., sensitivity or subgroup analyses, meta-regression analyses*, alternative network geometries studied, alternative choice of prior distributions for Bayesian analyses,* and so forth).	7<br>eFigure 4
**DISCUSSION**			
Summary of evidence	24	Summarize the main findings, including the strength of evidence for each main outcome; consider their relevance to key groups (e.g., healthcare providers, users, and policy-makers).	8-11
Limitations	25	Discuss limitations at study and outcome level (e.g., risk of bias), and at review level (e.g., incomplete retrieval of identified research, reporting bias). *Comment on the validity of*	11, 12

		the assumptions, such as transitivity and consistency. Comment on any concerns regarding network geometry (e.g., avoidance of certain comparisons).	
Conclusions	26	Provide a general interpretation of the results in the context of other evidence, and implications for future research.	12
**FUNDING**			
Funding	27	Describe sources of funding for the systematic review and other support (e.g., supply of data); role of funders for the systematic review. This should also include information regarding whether funding has been received from manufacturers of treatments in the network and/or whether some of the authors are content experts with professional conflicts of interest that could affect use of treatments in the network.	13

Abbreviation: PICOS = population, intervention, comparators, outcomes, study design.

* Text in italics indicates wording specific to reporting of network meta-analyses that has been added to guidance from the PRISMA statement.

† Authors may wish to plan for use of appendices to present all relevant information in full detail for items in this section.

Reference:

Hutton B, Salanti G, Caldwell DM et al. The PRISMA extension statement for reporting of systematic reviews incorporating network meta-analyses of health care interventions: checklist and explanations. Ann Intern Med 2015; 162: 777-784.

eTable 2. CHEERS Checklist.

Section/item	Item No	Recommendation	Reported on page No
**Title and abstract**			
Title	1	Identify the study as an economic evaluation or use more specific terms such as “cost-effectiveness analysis”, and describe the interventions compared.	1
Abstract	2	Provide a structured summary of objectives, perspective, setting, methods (including study design and inputs), results (including base case and uncertainty analyses), and conclusions.	2
**Introduction**			
Background and objectives	3	Provide an explicit statement of the broader context for the study.	3-4
		Present the study question and its relevance for health policy or practice decisions.	
**Methods**			
Target population and subgroups	4	Describe characteristics of the base case population and subgroups analysed, including why they were chosen.	5
Setting and location	5	State relevant aspects of the system(s) in which the decision(s) need(s) to be made.	6
Study perspective	6	Describe the perspective of the study and relate this to the costs being evaluated.	6
Comparators	7	Describe the interventions or strategies being compared and state why they were chosen.	5
Time horizon	8	State the time horizon(s) over which costs and consequences are being evaluated and say why appropriate.	6
Discount rate	9	Report the choice of discount rate(s) used for costs and outcomes and say why appropriate.	6
Choice of health outcomes	10	Describe what outcomes were used as the measure(s) of benefit in the evaluation and their relevance for the type of analysis performed.	6
Measurement of effectiveness	11a	*Single study-based estimates:* Describe fully the design features of the single effectiveness study and why the single study was a sufficient source of clinical effectiveness data.	6

	11b	*Synthesis-based estimates:* Describe fully the methods used for identification of included studies and synthesis of clinical effectiveness data.	
Measurement and valuation of preference based outcomes	12	If applicable, describe the population and methods used to elicit preferences for outcomes.	5-6
Estimating resources and costs	13a	*Single study-based economic evaluation:* Describe approaches used to estimate resource use associated with the alternative interventions. Describe primary or secondary research methods for valuing each resource item in terms of its unit cost. Describe any adjustments made to approximate to opportunity costs.	6
	13b	*Model-based economic evaluation:* Describe approaches and data sources used to estimate resource use associated with model health states. Describe primary or secondary research methods for valuing each resource item in terms of its unit cost. Describe any adjustments made to approximate to opportunity costs.	
Currency, price date, and conversion	14	Report the dates of the estimated resource quantities and unit costs. Describe methods for adjusting estimated unit costs to the year of reported costs if necessary. Describe methods for converting costs into a common currency base and the exchange rate.	6
Choice of model	15	Describe and give reasons for the specific type of decision-analytical model used. Providing a figure to show model structure is strongly recommended.	6 and eFigure 2
Assumptions	16	Describe all structural or other assumptions underpinning the decision-analytical model.	7
Analytical methods	17	Describe all analytical methods supporting the evaluation. This could include methods for dealing with skewed, missing, or censored data; extrapolation methods; methods for pooling data; approaches to validate or make adjustments (such as half cycle corrections) to a model; and methods for handling population heterogeneity and uncertainty.	7
**Results**			

Study parameters	18	Report the values, ranges, references, and, if used, probability distributions for all parameters. Report reasons or sources for distributions used to represent uncertainty where appropriate. Providing a table to show the input values is strongly recommended.	5-7 (Table 1)
Incremental costs and outcomes	19	For each intervention, report mean values for the main categories of estimated costs and outcomes of interest, as well as mean differences between the comparator groups. If applicable, report incremental cost-effectiveness ratios.	7 (Table 2)
Characterising uncertainty	20a	*Single study-based economic evaluation:* Describe the effects of sampling uncertainty for the estimated incremental cost and incremental effectiveness parameters, together with the impact of methodological assumptions (such as discount rate, study perspective).	6, 8
	20b	*Model-based economic evaluation:* Describe the effects on the results of uncertainty for all input parameters, and uncertainty related to the structure of the model and assumptions.	
Characterising heterogeneity	21	If applicable, report differences in costs, outcomes, or cost-effectiveness that can be explained by variations between subgroups of patients with different baseline characteristics or other observed variability in effects that are not reducible by more information.	8
**Discussion**			
Study findings, limitations, generalisability, and current knowledge	22	Summarise key study findings and describe how they support the conclusions reached. Discuss limitations and the generalisability of the findings and how the findings fit with current knowledge.	8-12
**Other**			
Source of funding	23	Describe how the study was funded and the role of the funder in the identification, design, conduct, and reporting of the analysis. Describe other non-monetary sources of support.	13
Conflicts of interest	24	Describe any potential for conflict of interest of study contributors in accordance with journal policy. In the absence of a journal policy, we recommend authors comply with International Committee of Medical Journal Editors recommendations.	13

A good template page for CHEERS Checklist is as follows: http://www.ispor.org/TaskForces/EconomicPubGuidelines.asp.

Reference:

Husereau D, Drummond M, Petrou S, et al. Consolidated health economic evaluation reporting standards (CHEERS) — Explanation and elaboration: A report of the ISPOR health economic evaluations publication guidelines good reporting practices task force. Value Health 2013;16:231-50.

eTable 3. Search Strategy.

Database	Keywords
**Pubmed**	
**(1)**	"apatinib"[Title/Abstract] OR "lenvatinib"[Title/Abstract] OR "sorafenib"[Title/Abstract] OR "vascular endothelial growth factor receptor inhibitors"[Title/Abstract] OR "VEGFR inhibitors"[Title/Abstract] OR OR "tyrosine kinase inhibitors"[Title/Abstract] OR "targeted therapy"[Title/Abstract] OR "molecular targeted therapy"[MeSH Terms] OR "nivolumab"[Title/Abstract] OR "pembrolizumab"[Title/Abstract] OR "ipilimumab" [Title/Abstract] OR "atezolizumab"[Title/Abstract] OR "camrelizumab"[Title/Abstract] OR "cemiplimab"[Title/Abstract] OR "durvalumab"[Title/Abstract] OR "toripalimab"[Title/Abstract] OR "tislelizumab"[Title/Abstract] OR "PD-1"[Title/Abstract] OR "PD-L1"[Title/Abstract] OR "anti-PD-1"[Title/Abstract] OR "anti-PD-L1"[Title/Abstract] OR "Immune checkpoint inhibitor"[Title/Abstract] OR "ICIs "[Title/Abstract] OR "programmed cell death 1 receptor/antagonists and inhibitors"[MeSH Terms] OR "programmed cell death 1 receptor antagonists and inhibitors"[Title/Abstract] OR "programmed cell death 1 receptor antagonist"[Title/Abstract] OR "programmed cell death 1 receptor inhibitor"[Title/Abstract] OR "immunotherapy"[Title/Abstract]
**(2)**	"radioiodine refractory differentiated thyroid cancer"[Title/Abstract] OR "radioiodine-refractory differentiated thyroid cancer"[Title/Abstract] OR "Radioactive iodine-refractory differentiated thyroid cancer"[Title/Abstract] OR RR-DTC[Title/Abstract]
**(3)**	"clinical trials as topic"[MeSH Terms] OR "clinical trial"[Publication Type] OR "phase III"[Title/Abstract] OR "phase 3"[Title/Abstract] OR "clinical trials"[Title/Abstract]
**(4)**	("2010/01/01"[Date - Publication] : "2022/03/20"[Date - Publication])
**(5)**	"review"[Article type] OR "meta"[Title] OR "meta-analysis"[Title] OR "protocol"[Title]
**(5)**	(1) AND (2) AND (3) AND (4) NOT (5)
**Embase**	
**(1)**	(apatinib OR lenvatinib OR sorafenib OR ‘vascular endothelial growth factor receptor inhibitors’ OR ‘VEGFR inhibitors’ OR ‘tyrosine kinase inhibitors’ OR ‘targeted therapy’ OR ‘molecular targeted therapy’ OR nivolumab OR pembrolizumab OR ipilimumab OR atezolizumab OR camrelizumab OR cemiplimab OR durvalumab OR toripalimab OR tislelizumab OR tislelizumab OR PD-1 OR PD-L1 OR anti-PD-1 OR anti-PD-1 OR anti-PD-L1 OR 'immune checkpoint inhibitors' OR ICIs OR 'programmed cell death 1 receptor/antagonists and inhibitors' OR 'programmed cell death 1 receptor antagonists and inhibitors'

	OR 'programmed cell death 1 receptor antagonist' OR 'programmed cell death 1 receptor inhibitor' OR immunotherapy):ti,ab,kw
**(2)**	(‘radioiodine refractory differentiated thyroid cancer’ OR ‘radioiodine-refractory differentiated thyroid cancer’ OR ‘Radioactive iodine-refractory differentiated thyroid cancer’ OR RR-DTC):ti,ab,kw
**(3)**	trial/exp OR 'clinical trials'/exp OR 'phase 3 clinical trial'/exp OR 'phase III clinical trial'/exp
**(4)**	(1) AND (2) AND (3) AND [01-01-2010]/sd NOT [20-03-2022]/sd
**Cochrane**	
**(1)**	(apatinib OR lenvatinib OR sorafenib OR ‘vascular endothelial growth factor receptor inhibitors’ OR ‘VEGFR inhibitors’ OR OR ‘tyrosine kinase inhibitors’ OR ‘targeted therapy’ OR ‘molecular targeted therapy’ OR nivolumab OR pembrolizumab OR ipilimumab OR atezolizumab OR camrelizumab OR OR cemiplimab OR durvalumab OR toripalimab OR tislelizumab OR tislelizumab OR PD-1 OR PD-L1 OR anti-PD-1 OR anti-PD-1 OR anti-PD-L1 OR 'immune checkpoint inhibitors' OR ICIs OR 'programmed cell death 1 receptor/antagonists and inhibitors' OR 'programmed cell death 1 receptor antagonists and inhibitors' OR 'programmed cell death 1 receptor antagonist' OR 'programmed cell death 1 receptor inhibitor' OR immunotherapy) AND (‘radioiodine refractory differentiated thyroid cancer’ OR ‘radioiodine-refractory differentiated thyroid cancer’ OR ‘Radioactive iodine-refractory differentiated thyroid cancer’ OR RR-DTC) AND ('clinical trials as topic' OR trial) NOT ("conference" OR "review") (Publication date: Between Jan 1, 2020 and Marh 20, 2022)
**Web of science**	
**(1)**	TS=(apatinib OR lenvatinib OR sorafenib OR ‘vascular endothelial growth factor receptor inhibitors’ OR ‘VEGFR inhibitors’ OR ‘tyrosine kinase inhibitors’ OR ‘targeted therapy’ OR ‘molecular targeted therapy’ OR nivolumab OR pembrolizumab OR ipilimumab OR atezolizumab OR camrelizumab OR cemiplimab OR durvalumab OR toripalimab OR tislelizumab OR tislelizumab OR PD-1 OR PD-L1 OR anti-PD-1 OR anti-PD-1 OR anti-PD-L1 OR 'immune checkpoint inhibitors' OR ICIs OR 'programmed cell death 1 receptor/antagonists and inhibitors' OR 'programmed cell death 1 receptor antagonists and inhibitors' OR 'programmed cell death 1 receptor antagonist' OR 'programmed cell death 1 receptor inhibitor' OR immunotherapy)
**(2)**	TS=("radioiodine refractory differentiated thyroid cancer" OR "radioiodine-refractory differentiated thyroid cancer" OR "Radioactive iodine-refractory differentiated thyroid cancer" OR RR-DTC)
**(3)**	TS=("clinical trials as topic"OR "trial" OR "phase III" OR "phase 3" OR "clinical trials")
**(4)**	TS=("conference" OR "review" OR "meta")
**(5)**	(1) AND (2) AND (3) NOT (4) (Publication date：2010-01-01 to 2022-03-20)

eTable 4. Drug dose and cost.

Drug	Dose	Infusion Timing	Unit costs($)
Apatinib	Apatinib, 500mg	500 mg was administered orally once daily	2.6454 per 1mg
Lenvatinib	Lenvatinib, 24mg	500 mg was administered orally once daily	171.1503 per 1mg

eTable 5. Patient Baseline Characteristics.

Characteristic	Apatinib group	Lenvatinib group
**Median age (range), years**	56 (31-75)	61 (28-80)
**Sex**		
Male	58.7%	55.3%
Female	41.3%	44.7%
**Chinese**	100%	100%
**ECOG performance status**		
0	52.2%	55.3%
1	43.5%	39.8%
2	4.3%	4.9%
**Histologic subtype**		
Papillary	80.4%	80.6%
Follicular	19.6%	19.4%
**No Iodine-131 uptake**	65.2%	58.3%
**Prior targeted therapy**	10.9%	25.2%
**Lung metastasis**	82.6%	88.3%

Abbreviation: ECOG=Eastern Cooperative Oncology Group.

eTable 6. Summary of statistical goodness-of-fit of K-M curve.

	Exponential	Weibull	Gompertz	Log-logistic	Log-normal
**Apatinib OS curve**					
AIC	8.3998	**6.7415**	8.4757	8.3797	8.3334
BIC	9.9450	**7.5141**	10.0209	9.9249	9.8786
**Lenvatinib OS curve**					
AIC	7.2177	**5.3160**	7.2424	7.2122	7.1950
BIC	8.1875	**5.8009**	8.2123	8.1820	8.1648
**Apatinib PFS curve**					
AIC	19.9592	**17.4968**	20.3141	19.6267	19.6044
BIC	21.3753	**18.2048**	21.7302	21.0428	21.0205
**Lenvatinib PFS curve**					
AIC	15.0331	**13.0960**	15.0931	14.9391	14.9114
BIC	16.1630	**13.6609**	16.2230	16.0690	16.0413

Abbreviation: OS, overall survival; PFS, progression-free survival; AIC, Akaike's information criterion; BIC, Bayesian information criterion.

Weibull distributions are flexible and widely used were matched to the number of patients in the three states over time, as its can monotonically increase or decrease the hazard function, it is suitable for estimating the event that occurs in the early follow-up work period. And according to AIC, BIC and the visual fits of the curves (eFigure 3 in the supplementary material), we choose the Weibull distributions as the most reasonable parametric survival model.

eTable 7. Characteristics of RCTs included in the study.

Study	Phase	Trial name	Total sample size	Drug	Combination	Sample size	Control	Sample size	Median OS (months)	HR for OS (95% CI)	Median PFS (months)	HR for PFS (95% CI)	Patients with grade 3 or higher AEs, n (%)
H Luo, 2021	III	ESCORT-1st	596	Camrelizumab PD-1	Paclitaxel and Cisplatin	298	Placebo with Paclitaxel and Cisplatin	298	15.3 vs 12.0	0.70 (0.56 to 0.88)	6.9 vs 5.6	0.56 (0.46 to 0.68)	189 (63.4) vs 201 (67.7)
Z Wang, 2022	III	JUPITER-06	514	Toripalimab PD-1	Paclitaxel and Cisplatin	257	Placebo with Paclitaxel and Cisplatin	257	17.0 vs 11.0	0.58 (0.43 to 0.78)	5.7 vs 5.5	0.58 (0.46 to 0.74)	188 (73.2) vs 180 (70.0)

Abbreviation: OS ,Overall Survival; PFS, progression-freesurvival; CI, confidence interval; AEs, Adverse Events.

eTable 8. Results of subgroup analyses.

Subgroup	Size (%)		HR for PFS（95% CI）	Change in cost, $[a]	Change in QALYs[a]	ICER, $/QALY	Cost-effectiveness probability of apatinib, % at WTP of $37,653/QALY
	Apatinib	Lenvatinib					
**Age, years**							
≤65	83%	71%	1.47 (0.60 to 3.58)	-11,233	0.803	Dominated[b]	100%
＞65	17%	29%	2.78 (0.52 to 14.77)	13,579	1.002	13,547	55%
**Sex**							
Male	41%	55%	1.26 (0.42 to 3.80)	-18,362	0.746	Dominated[b]	100%
Female	59%	45%	2.46 (0.80 to 7.51)	9,245	0.968	9,555	85%
**Histology**							
Papillary	80%	86%	1.68 (0.71 to 4.00)	-5,456	0.850	Dominated[b]	100%
Follicular[c]	20%	19%	NA	NA	NA	NA	NA

[a] HR for PFS represents the HR of apatinib vs lenvatinib for PFS; change in cost and change in QALYs represent the results of apatinib minu lenvatinib.
b Apatinib showed higher effectiveness and lower cost, as compared with the lenvatinib.
[c] HR unavailable in this subgroup.
Abbreviation: PFS, progression-freesurvival; CI, confidence interval; QALYs, quality-adjusted life-years; ICER, incremental cost-effectiveness ratio;WTP, willing-to-pay.
